# Multiple lymphomatous polyposis with diffuse involvement of the gastrointestinal tract: a case report

**DOI:** 10.1186/1471-2482-13-S1-A7

**Published:** 2013-09-16

**Authors:** Giovanni Cestaro, Michele De Rosa, Chiara Vitiello, Maurizio Gentile

**Affiliations:** 1 Dipartimento di Medicina Clinica e Chirurgia – U.O.C. di Chirurgia Generale, Università Federico II – via Pansini 5 Napoli, Italy

## Introduction

The gastrointestinal tract is the predominant site of appearance of extranodal non-Hodgkin lymphomas. Multiple lymphomatous polyposis (MLP) is a type of manifestation of mantle cell lymphoma (MCL) and it is characterized by multiple polypoid lesions involving long segments of the gastrointestinal tract and it accounts for only approximately 1-2% of non Hodgkin lymphomas.

## Methods

A 78 years old patient was admitted to our Department of General Surgery with rectal bleeding, abdominal pain and weight loss. Multiple lymphomatous polyposis was detected on the endoscopic evaluations, i.e. upper GI endoscopy and colonoscopy. Abdominal and pelvis TC revealed anomalies of the rectum wall, pelvic lymphadenopaty and two liver metastasis. Abdominal and pelvis RM showed lombo-aortic lymphadenopaty and massive thickening of the rectum. Total body PET-TC detected rachis involvement, specifically at D1, L2 and L3 vertebrae, and an intense signal of the whole ileum.

## Results

Histopathologic study of biopsies from gastrointestinal specimens showed a lymphoid infiltrate. Immunoistochemical evaluation confirmed that the lymphoma was positive for CD20 and CYCLIN D1 and negative for CD10, CD23 and CD5, features suitable with diagnosis of non-Hodgkin’s mantle lymphoma. Bone narrow biopsy was negative for lymphoid proliferation. The patient was sent to Department of Hematology to receive cycles of chemioterapy.

## Conclusions

MCL is an uncommon entity of primary GI NHL with particular clinic, morphological and immunophenotypic features. Clinically, patients with a diagnosis of MCL are often elderly adults with a male predominance and present a disease in an advanced stage. Abdominal pain, diarrhea, hematochezia and organomegaly are the most common presenting manifestations. Because this lymphoma occurs in the elderly population, stem cell transplantation is not possible. GI lymphomatous polyposis is a rare disease, but in all elderly patients with a widespread polyposis of the GI tract a diagnosis of lymphoma must be considered.
